# Paediatric Behçet's disease presenting with recurrent papillitis and episcleritis: a case report

**DOI:** 10.1186/1752-1947-5-81

**Published:** 2011-02-25

**Authors:** Fulvio Parentin, Loredana Lepore, Ingrid Rabach, Stefano Pensiero

**Affiliations:** 1 Department of Surgery, Ophthalmology Unit, Institute for Maternal and Child Health Burlo Garofolo, Via dell'Istria, 65/1, Trieste I-34100, Italy; 2 Department of Paediatrics, Rheumatology Unit, Institute for Maternal and Child Health Burlo Garofolo, Via dell'Istria, 65/1, Trieste I-34100, Italy

## Abstract

**Introduction:**

Behçet's disease is a chronic multisystem vasculitis characterized by mucocutaneous, articular, neurological, gastrointestinal and ophthalmological lesions. Ocular involvement is mainly represented by recurrent uveitis, especially posterior uveitis; however, iridocyclitis, retinal and choroidal vasculitis, optic neuritis and retinal vascular occlusion can also occur.

**Case presentation:**

A 12-year-old Caucasian boy with a history of recurrent buccal aphthosis and nonspecific gastrointestinal symptoms was admitted to our hospital with blurred vision associated with acute episcleritis and papillitis. The patient's pathergy test was positive, suggesting a diagnosis of Behçet's disease. Corticosteroid and cyclosporine therapy was started, but further episodes were noted in both eyes. The patient was then switched to intravenous infliximab, with complete resolution of the inflammation after the second infusion.

**Conclusion:**

Episcleritis and papillitis should be added to the list of uncommon manifestations of pediatric Behçet's disease. Infliximab is an effective, new therapeutic approach for Behçet's disease that is refractory to the conventional corticosteroid and immunosuppressive therapy.

## Introduction

Behçet's disease is a chronic multisystem vasculitis characterized by mucocutaneous, articular, neurological, gastrointestinal and ophthalmological lesions [1]. The hallmark of ocular involvement is recurrent uveitis, especially posterior uveitis; however, iridocyclitis, retinal and choroidal vasculitis, optic neuritis and retinal vascular occlusion can also occur [2]. Other ocular manifestations, such as scleritis, are very unusual and are described only among adult patients [3,4]. We report an exceedingly rare manifestation of childhood-onset Behçet's disease occurring together with recurrent and simultaneous episcleritis and papillitis.

## Case presentation

**Figure 1:**
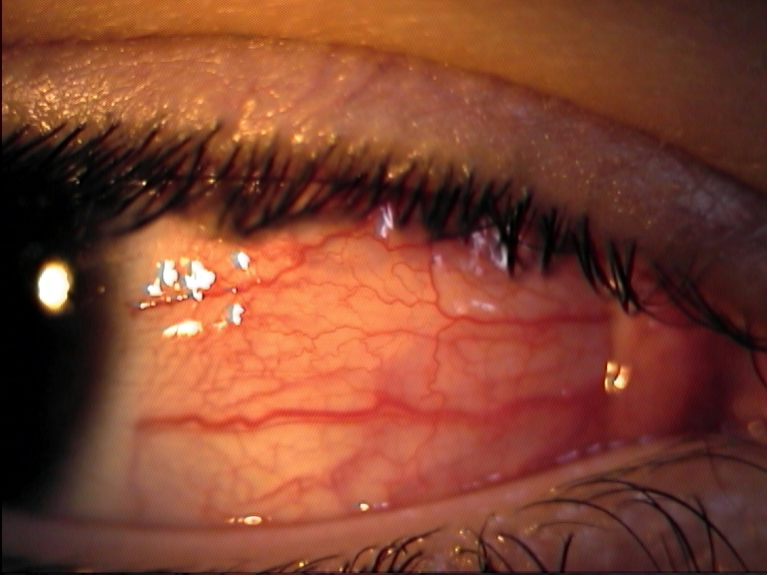
**Diffuse right episcleritis with injection in the superficial episcleral vessels**.

**Figure 2:**
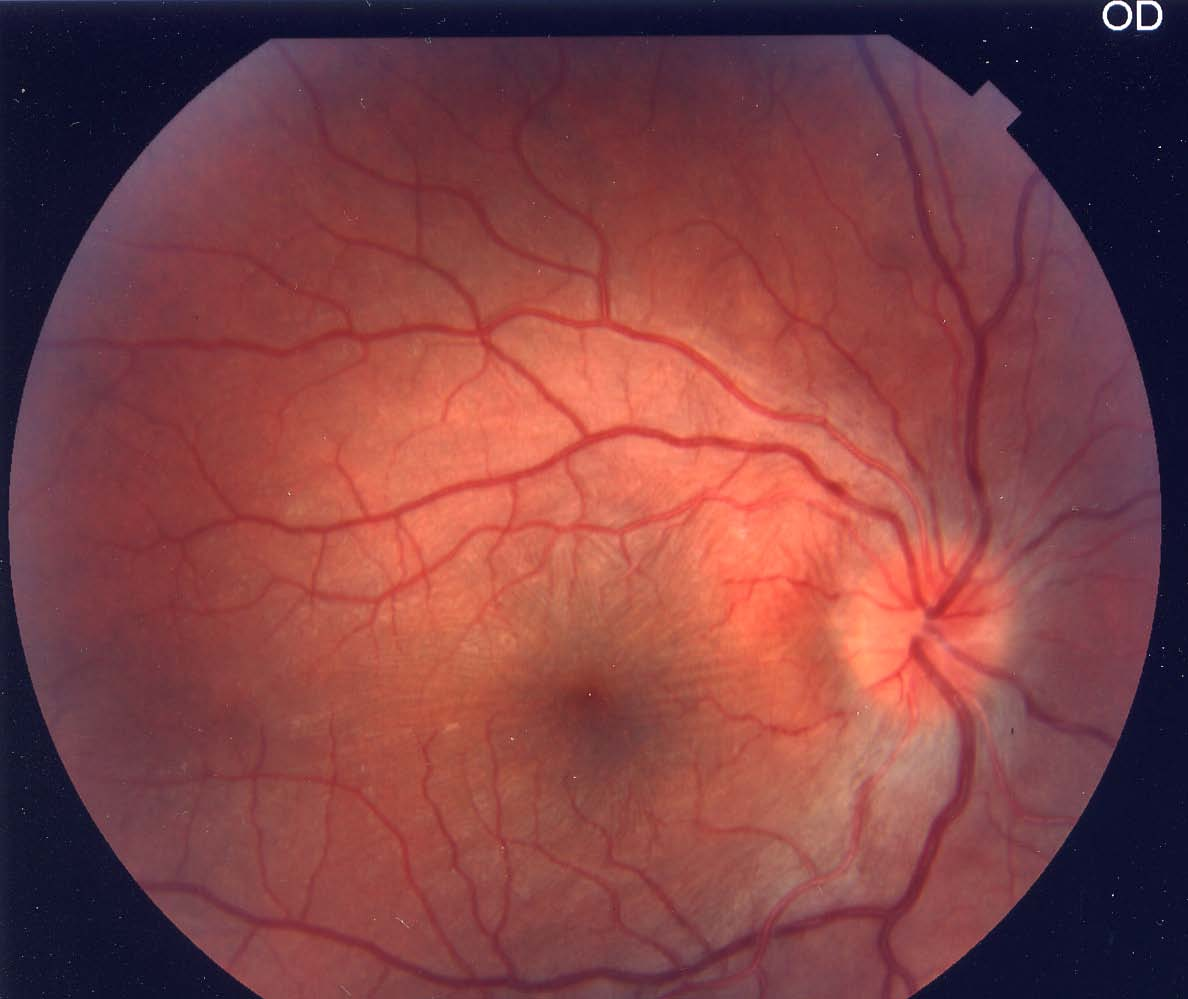
**Hyperemic optic disc with blurred margins (color fundus photograph)**.

**Figure 3:**
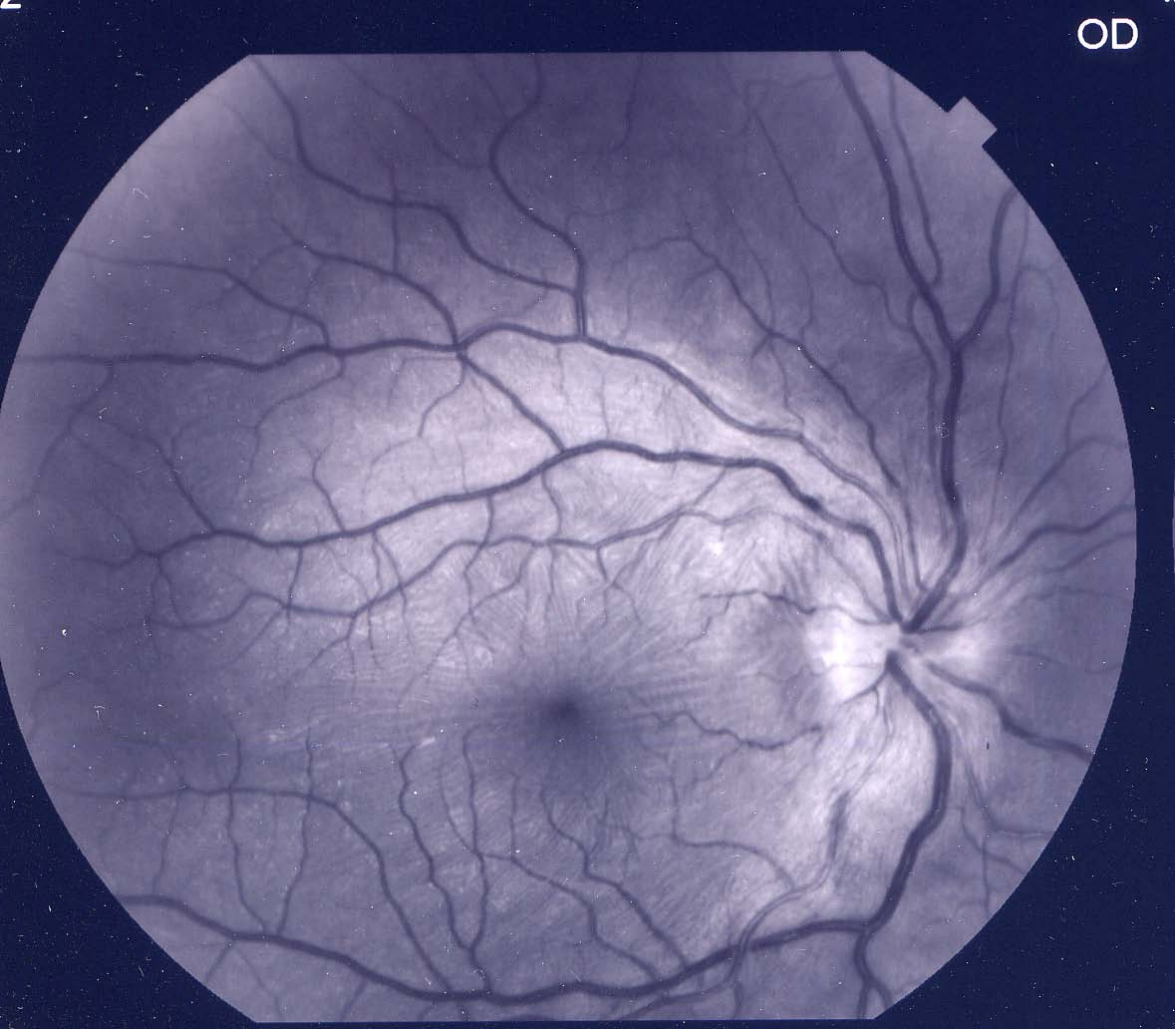
**Hyperemic optic disc with blurred margins (red-free fundus photograph)**.

A 12-year-old Caucasian boy was admitted with painful redness in the conjunctiva and acutely blurred vision in his right eye. He referred a history of recurrent buccal aphthosis and nonspecific gastrointestinal symptoms, such as abdominal pain and diarrhea. At admission, decreased visual acuity in the right eye (20/200) was observed. A slit lamp examination showed diffuse and painful episcleritis with injection in the superficial episcleral vessels (Figure 1); the fundus examination revealed a right hyperemic disc with blurred margins (Figures 2 and 3). Inflammatory markers were increased (erythrocyte sedimentation rate, 31 mm/1 h; C-reactive protein, 0.7 mg/L). The laboratory tests for infectious diseases, C3 and C4, rheumatoid factor, anti-double-stranded DNA (dsDNA) and anti-Smith antigen antineutrophil cytoplasmic antibodies were negative. Human leukocyte antigen typing was negative for B51, showing A02, A68, B44, B39, DRB1 and DRB3. A pathergy test, performed using an intradermal 21-gauge needle puncture on the skin of the forearm, was positive. According to the criteria of the International Study Group for Behçet's Disease, we diagnosed right optic neuritis and episcleritis secondary to Behçet's disease and commenced corticosteroid therapy with oral prednisone 1 mg/kg and dexamethasone eyedrops six times daily in the right eye. The patient's fundal and conjunctival appearance returned to normal within one week, with his visual acuity returning to 20/20. Therapy was therefore stopped. After this initial manifestation, the disease relapsed four times in the following six months in both eyes. The patient was therefore commenced on oral cyclosporine 100 mg twice daily, but another two episodes were noted, and the therapy was stopped after four months. He was then switched to endovenous infliximab, a chimeric anti-tumor necrosis factor-α (anti-TNF-α) monoclonal antibody, in an attempt to control the disease. Infusions of 5 mg/kg infliximab were administered at zero, two and six weeks and then at intervals of eight weeks. There was a remarkable response soon after the first infusion and a complete resolution of the inflammation after the second infusion. The patient remains well one year later and continues to enjoy remission on continued therapy with infliximab at intervals of eight weeks.

## Discussion

Childhood-onset Behçet's disease is uncommon, accounting for 3% to 7% of all cases [2]. Scleritis may be a harbinger of systemic autoimmune inflammatory diseases. Few publications have reported an association between Behçet's disease and scleral inflammation, and only among adult patients. Episcleritis is a benign inflammation confined to the episcleral tissue; patients with episcleritis usually complain of mild pain. In 2004, Dursun *et al. *[3] reported a case of systemic Behçet's disease with anterior necrotizing scleritis, lateral rectus myositis and recurrent retrobulbar optic neuritis. Nodular and diffuse scleritis is characterized by edema and injection in both the superficial and deep episcleral vessels, and pain is usually severe and deep-seated. Finally, scleromalacia perforans is a necrotizing scleritis, relatively asymptomatic and without inflammation. In 2005 Sakellariou *et al. *[4] reported the unique association between Behçet's disease and scleromalacia perforans.

Optic neuritis is a rare manifestation of neuro-Behçhet's disease. A search of MEDLINE retrieved only one case report of inflammatory optic nerve involvement in the pediatric age group [5]. The diagnosis in children is difficult, as the disease is uncommon and clinically resembles other diseases, such as multiple sclerosis. Another important differential diagnosis is central venous sinus thrombosis, which is well described in Behçet's disease and is usually characterized by severe headache and deterioration in general condition.

Infliximab, a chimeric monoclonal antibody to TNF-α, was developed and used to treat systemic inflammatory disorders such as rheumatoid arthritis and Crohn's disease. Proinflammatory cytokines, including TNF-α, are known to be elevated in active Behçet's disease, suggesting that anti-TNF-α therapy might be effective. Clinically, significant improvement of various Behçet's disease manifestations with infliximab therapy has been reported in the literature [6,7]. A recent study demonstrated that the effectiveness of infliximab on the ocular inflammation in Behçet's disease correlates with the infliximab serum concentrations [8].

## Conclusion

First, our report underlines that episcleritis and papillitis without uveitis should be added to the list of uncommon manifestations of pediatric Behçet's disease. Second, infliximab seems to be an effective drug for the management of Behçet's disease that is refractory to the conventional corticosteroid and immunosuppressive therapy. The selection of optimal dose and frequency of infusion required standardization for our patient.

## Competing interests

The authors declare that they have no competing interests.

## Informed consent

Written informed consent was obtained from the patient's legal guardian for publication of this case report and accompanying images. A copy of the written consent is available for review by the Editor-in-Chief of this journal.

## Authors' contributions

FP and SP interpreted the ophthalmological manifestation of the disease. FP was also the major contributor in writing the manuscript. LL and IR analyzed the patient data regarding the systemic manifestation of the disease. They also provided support in administering the systemic immunosuppressive and biological therapy. All authors read and approved the final manuscript.
